# Molecular Regulation of Antioxidant Melatonin Biosynthesis by Brassinosteroid Acting as an Endogenous Elicitor of Melatonin Induction in Rice Seedlings

**DOI:** 10.3390/antiox11050918

**Published:** 2022-05-06

**Authors:** Ok Jin Hwang, Kyoungwhan Back

**Affiliations:** Department of Biotechnology, College of Agriculture and Life Sciences, Chonnam National University, Gwangju 61186, Korea; smilax@jnu.ac.kr

**Keywords:** epibrassinolide, gibberellin, melatonin, *DWARF4*, *DWARF11*, *RAV-Like1*, *Gα*, transgenic rice

## Abstract

Gibberellic acid (GA) was recently shown to induce melatonin synthesis in rice. Here, we examined whether brassinosteroids (BRs) also induce melatonin synthesis because BRs and GA show redundancy in many functions. Among several plant hormones, exogenous BR treatment induced melatonin synthesis by twofold compared to control treatment, whereas ethylene, 6-benzylaminopurine (BA), and indole-3-acetic acid (IAA) showed negligible effects on melatonin synthesis. Correspondingly, BR treatment also induced a number of melatonin biosynthetic genes in conjunction with the suppression of melatonin catabolic gene expression. Several transgenic rice plants with downregulated BR biosynthesis-related genes, such as *DWARF4*, *DWARF11*, and *RAV-Like1* (*RAVL1*), were generated and exhibited decreased melatonin synthesis, indicating that BRs act as endogenous elicitors of melatonin synthesis. Notably, treatment with either GA or BR fully restored melatonin synthesis in the presence of paclobutrazol, a GA biosynthesis inhibitor. Moreover, exogenous BR treatment partially restored melatonin synthesis in both *RAVL1* and *Gα* RNAi transgenic rice plants, whereas GA treatment fully restored melatonin synthesis comparable to wild type in *RAVL1* RNAi plants. Taken together, our results highlight a role of BR as an endogenous elicitor of melatonin synthesis in a GA-independent manner in rice plants.

## 1. Introduction

Melatonin is a multifunctional molecule commonly found in bacteria, archaea, plants, and animals [1,2]. In plants, melatonin is thought to be a master regulator of a diverse array of physiological functions, with roles not only in growth and development but also in defense responses against many biotic and abiotic stressors [3,4]. Many of these functions of melatonin, particularly stress tolerance responses, are derived from its intrinsic activity as a potent antioxidant that regulates redox balance in plants [1]. For instance, it is known that one molecule of melatonin scavenges up to 10 molecules of reactive oxygen species or reactive nitrogen species [5], as well as the induction of many antioxidant enzymes such as catalase and superoxide dismutase [1,3]. In addition, melatonin acts as a signaling molecule in plants to activate a mitogen-activated protein kinase (MAPK) pathway followed by the induction of many genes involved in defense against pathogens [6,7], protein quality control [8,9], stomatal closure [10], and yield increase [11,12]. Consistent with its pleiotropic roles, the mode of action of melatonin in plants is closely associated with many plant hormones by either upregulating or downregulating corresponding genes [13]. For example, the suppression of melatonin synthesis leads to decreases in levels of brassinosteroids (BR) in several RNAi transgenic rice plants with BR-deficient phenotypes, such as semi-dwarf with erect leaves [14]. Notably, melatonin-rich transgenic rice plants produce larger seeds associated with increased cytokinin synthesis [12]. In addition, exogenous melatonin treatment also promotes primary root growth in *Arabidopsis thaliana* in an indole-3-acetic acid (IAA)-dependent manner [15]. In contrast to the direct effects of exogenous melatonin application, *A*. *thaliana SNAT1* and *SNAT2* knockout mutants show delayed flowering due to significantly decreased expression of *ent-kaurene synthase*, the key gene for GA synthesis, due to decreased starch synthesis [8,16,17]. These data clearly indicate that the physiological functions of melatonin are mediated by interactions with many plant hormones.

Although it has also been reported that melatonin-treated plants have altered levels of many hormones in response to many stress challenges to protect plants from stress-related damage [13,18,19], the roles of plant hormones in the regulation of melatonin synthesis remain poorly understood. Recently, we reported that gibberellic acid (GA) acts as an endogenous elicitor to induce melatonin synthesis in rice seedlings and immature seeds, pointing to a positive correlation between GA and melatonin synthesis [20]. On the basis of our previous findings, we examined the role of BR in melatonin synthesis because BR and GA have redundant functions in many developmental processes. Moreover, BRs induce GA biosynthesis and promote GA signaling [21,22].

BRs are steroid hormones that control multiple physiological processes throughout the entire life cycle of the plant, including cell elongation, dark-induced seedling growth, leaf angle, and seed size, as well as defense responses against various abiotic and biotic stresses [23]. Among the 70 BRs found in plants, three (epibrassinolide, homobrassinolide, and brassinolide) have been widely used in pharmacological experiments [24]. BR biosynthesis starts from farnesyl pyrophosphate by squalene synthase followed by more than 12 enzymes, of which C-22 hydroxylation involving two redundant P450 enzymes, i.e., DWARF11 and DWARF4, seems to be the rate-limiting step for BR biosynthesis [25]. The transcription factor RAV-Like 1 (RAVL1) induces BR synthesis and increases BR sensitivity by binding the E-box elements of *BRASSINOSTEROID INSENSITIVE1* (*BRI1*), *ebisu dwarf* (*D2*), and *DWARF11* [26,27]. Upon BR treatment, the plasma-membrane-bound BR receptor BRI1 interacts with BRI1-ASSOCIATED RECEPTOR KINASE1 activating in several BR signaling kinases for the induction of downstream genes responsive to BR [24]. In addition to the BRI1 receptor pathway, G-protein-coupled signaling pathways are also associated with BR signaling, according to a previous study that found that a rice heterotrimeric G protein alpha subunit 1 (Gα) mutant showed decreased sensitivity to epibrassinolide, as indicated by effects on root growth and coleoptile elongation [28]. Interestingly, in another study, a rice mutant showed greatly reduced GA-induced α-amylase activity, indicating that Gα is also involved in GA signaling [29]. All mutants of BR synthesis and signaling mentioned above exhibit common phenotypes, such as semi-dwarfism and erect leaves.

In accordance with our hypothesis, BR also induced melatonin biosynthesis to levels comparable to GA treatment, indicating that both BR and GA are positively associated with the effects of melatonin synthesis on plant growth and development. Our findings may be applicable in agriculture to increase melatonin synthesis in plants and fruits by exogenously applying either GA or BR to produce melatonin-rich products with enhanced nutritional value that will be beneficial to human health.

## 2. Materials and Methods

### 2.1. Plant Growth Conditions

Dehusked rice (*Oryza sativa* cv. Dongjin) seeds which are currently cultivated in South Korea and widely used for rice transformation were sterilized with 2% NaOCl for 50 min, after which they were thoroughly rinsed with sterile distilled water and sown on half-strength Murashige and Skoog (MS) medium under cool daylight fluorescent lamps (60 μmol m^–2^ s^–1^) (Philips, Amsterdam, The Netherlands) under a 14 h light/10 h dark photoperiod at 28 °C/24 °C (day/night). The 7-day-old seedlings were used in further experiments.

### 2.2. Chemical Treatment

The 7-day-old rice seedlings in 50 mL polypropylene conical tubes containing 30 mL water were first treated with varying levels of epibrassinolide (BR; Sigma-Aldrich, St. Louis, MO, USA), ethephon (Sunmoon Green Science, Seoul, Korea), 6-benzylaminopurine (BA; Sigma-Aldrich), and indole-3-acetic acid (IAA; Sigma-Aldrich). The BR biosynthesis inhibitor brassinazole (100 µM; Sigma-Aldrich) and GA biosynthesis inhibitor paclobutrazol (10 µM; Sigma-Aldrich) were used to inhibit BR and GA biosynthesis, respectively. GA_3_ was purchased from Duchefa Biochemie (Harrlem, The Netherlands). BR, BA, and IAA were dissolved in 0.1% ethanol. The control (C) contained 0.1% ethanol in water, except for ethephon, where the control was water. Treatments were applied for 1 day under cool daylight fluorescent lamps (60 μmol m^–2^ s^–1^) (Philips) with a 14 h light/10 h dark photoperiod at 28 °C/24 °C (day/night), followed by treatment with 0.5 mM CdCl_2_ for 3 days under continuous light conditions (60 μmol photons m^–2^ s^–1^) for melatonin detection by high-performance liquid chromatography (HPLC). The upper parts of leaves and stems were harvested and stored in liquid nitrogen for further analyses.

### 2.3. Quantitative Real-Time Polymerase Chain Reaction (qRT-PCR) Analysis

Total RNA of rice plants was isolated using a NucleoSpin RNA Plant Kit (Macherey-Nagel, Düren, Germany). First-strand cDNA was synthesized from 2 µg of total RNA using RNA to cDNA using EcoDry^TM^ Premix (Takara Bio USA, Inc., Mountain View, CA, USA). qRT-PCR was performed in a Mic qPCR Cycler system (Biomolecular Systems, Queensland, VIC, Australia) with specific primers and the TB Green^®^ Premix Ex Taq^TM^ (Takara Bio Inc., Kusatsu, Shiga, Japan), as described previously [20]. The expression of genes was analyzed using Mic’s RQ software v2.2 (Biomolecular Systems) and normalized to *UBQ5*. qRT-PCR was performed with the following primer set: *DWARF4* forward 5′-GGA GAA GAA CAT GGA ATC AC-3′, *DWARF4* reverse 5′-GTA ATC TTG AAC GCG GAT ATG-3′, *DWARF11* forward 5′-TGA GGC ACT GAG ATG TGG-3′, *DWARF11* reverse 5′-AAG GTG ATG GAG GAA GAA-3′, *RAVL1* forward 5′-CGA CTT CCG CAA CAT CAA-3′, *RAVL1* reverse 5′- GGC ATC CGT AGC GAC AAT-3′, *D2* forward 5′-ATG TGA TAA CAG AGA CGC TGC GGT-3′, *D2* reverse 5′-TGG TGA CCA AGT GGT GAA GGA AGA-3′, *BRI1* forward 5′-CAG CTA CTT GGC TAT CTT GAA GCT CAG C-3′, *BRI1* reverse 5′-CCA TTC TTG TTG AAG GTG TAC TCC GTG C-3′, *UBQ5* forward 5′-CCG ACT ACA ACA TCC AGA AGG AG-3′, and *UBQ5* reverse 5′-AAC AGG AGC CTA CGC CTA AGC-3′. The primer information encoding genes involving melatonin biosynthesis and catabolism has been described previously [20].

### 2.4. Quantification of Melatonin

Frozen samples (0.1 g) were pulverized to a powder in liquid nitrogen using the TissueLyser II (Qiagen, Tokyo, Japan) and then extracted with 1 mL chloroform. The chloroform extracts were centrifuged for 10 min at 12,000× *g*, and then the supernatants (200 µL) were completely evaporated and dissolved in 0.1 mL of 40% methanol, and 10-µL aliquots were subjected to HPLC using a fluorescence detector system (Waters, Milford, MA, USA) as described previously [30]. Melatonin was eluted at about 31 min under this HPLC condition. All measurements were performed in triplicate.

### 2.5. Generation of DWARF4, DWARF11, and RAVL1 Suppression Transgenic Rice Plants by RNA Interference (RNAi)

The pTCK303 binary vector [31] was used to suppress rice *DWARF4* (GenBank accession number AB206579), DWARF11 (GenBank accession number AK106528), and RAVL1 (GenBank accession number Os04g0581400). In brief, an N-terminal 359 bp *DWARF4* cDNA fragment was amplified by reverse transcription-PCR using the following primer set: *DWARF4-F* 5′-ACT AGT ACC AGC GAG CTG CTC TTC-3′ (*Spe*I site underlined) and *DWARF4-R* 5′-GAG CTC GGT AGC TGC ACT CGA ACA-3′ (*Sac*I site underlined) with the cDNA templates synthesized from the total RNA from rice seedlings. As for *DWARF11*, a C-terminal 283 bp *DWAR11* cDNA was amplified with the following primer set: *DWARF11-F* 5′-ACT AGT GTT CAT TTG AAC CCC TTA-3′ (*Spe*I site underlined) and *DWARF11-R* 5′-GAG CTC AAG TGG CTC GAT TTC TAT-3′ (*Sac*I site underlined). Similarly, the N-terminal 300 bp length of *RAVL1* was amplified with the following primer set: *RAVL1-F* 5′-ACT AGT ATG GAG CAG GAG CAG GAT-3′ (*Spe*I site underlined) and *RAVL1-R* 5′-GAG CTC GTT CAG CTT CCC CAC GTC-3′ (*Sac*I site underlined). These PCR products were first subcloned into the T&A cloning vector (T&A:DWARF4, T&A:DWARF11, and T&A:RAVL1; RBC Bioscience, New Taipei City, Taiwan) for further cloning experiments. From these plasmids, the antisense *DWARF4, DWARF11*, and *RAVL1* inserts were acquired by *Sac*I and *Spe*I double digestion, whereas the sense *DWARF4, DWARF11*, and *RAVL1* inserts were prepared by *Kpn*I and *Bam*HI double digestion. The antisense fragments were first ligated into the pTCK303 vector followed by the sense fragments of three gene inserts. The resulting pTCK303:DWARF4, pTCK303:DWARF11, and pTCK303:RAVL1 RNAi binary vectors were independently transformed into *Agrobacterium tumefaciens* LBA4404 followed by *Agrobacterium*-mediated rice transformation with the embryogenic calli derived from rice seed (*Oryza sativa* cv. Dongjin) as previously described [11,32]. Transgenic rice plants suppressing Gα was generated previously and described in Hwang and Back [20].

### 2.6. Statistical Analysis

The data were analyzed using analysis of variance using IBM SPSS Statistics 23 software (IBM Corp. Armonk, NY, USA). Means with asterisks and different letters indicate significantly different values at *p* < 0.05, according to least significance difference (LSD) test. All data are presented as mean ± standard deviations.

## 3. Results

### 3.1. Effects of Various Hormone Treatments on Melatonin Synthesis in Rice Seedlings

**Figure 1:**
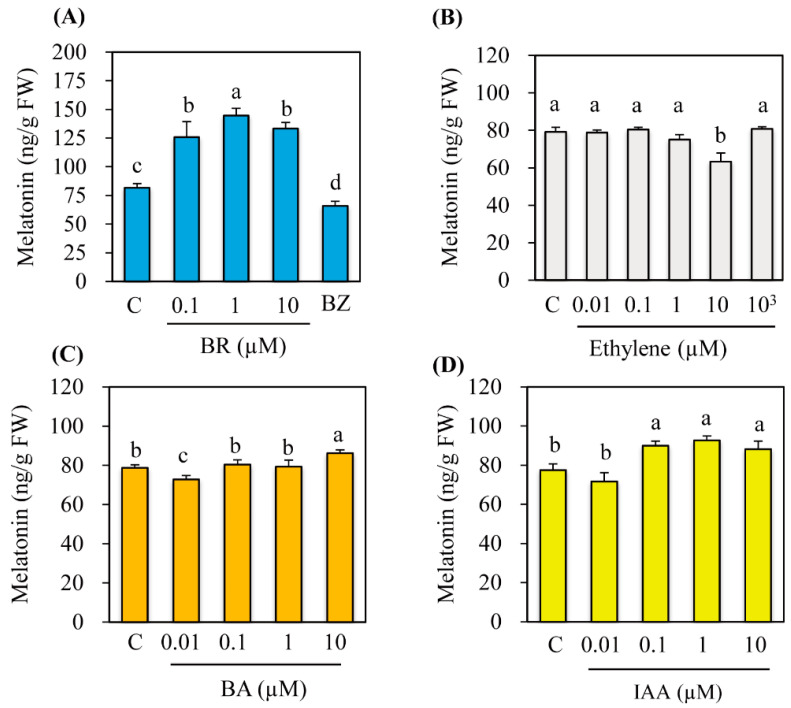
Effects of various hormones on melatonin synthesis. (**A**) Effects of BR on melatonin content. Brassinazole (BZ), a BR biosynthesis inhibitor, was used at a concentration of 100 μM. (**B**) Effects of ethylene on melatonin content. (**C**) Effects of 6-benzylaminopurine (BA) on melatonin content. (**D**) Effects of indole-3-acetic acid (IAA) on melatonin content. Seven-day-old rice seedlings were treated rhizosperically with varying levels of hormones independently for 24 h and transferred to new 50 mL conical tubes containing 0.5 mM cadmium for 3 days to induce melatonin synthesis. Different letters denote significant differences (*p* < 0.05) as determined by Tukey’s HSD post hoc test. C, water containing 0.1% ethanol. Ethephon that releases ethylene was used for ethylene treatment.

GA acts as an endogenous plant hormone to induce melatonin synthesis in rice [20]. Here, to determine whether other plant hormones can also induce melatonin synthesis, the roots of 7-day-old rice seedlings were independently treated with varying levels of four different hormones for 24 h, followed by cadmium treatment for induction of melatonin to facilitate the detection of melatonin by HPLC. As shown in Figure 1, ethylene was unable to induce melatonin synthesis, whereas BR showed a strong capacity to induce melatonin synthesis with peak melatonin accumulation at a concentration of 1 μM. The effect of BR was further confirmed by the application of brassinazole (BZ), a BR biosynthesis inhibitor. BZ treatment significantly inhibited melatonin synthesis, indicating that BR elicits melatonin synthesis, similar to GA [20]. In addition, cytokinin (BA) and IAA treatments slightly increased melatonin synthesis, but its levels were far lower than those of BR. It is puzzling that 10 μM ethylene treatment decreased melatonin synthesis that was not observed in either 1 μM or 100 μM treatment. The reason for this is unclear, but similar results were also observed in cytokinin (0.01 μM) and IAA (0.01 μM) treatments. In summary, these results clearly show that BR acts as a potent inducer of melatonin synthesis in rice seedlings.

### 3.2. Characterization of Genes Involved in Melatonin Biosynthesis and Catabolism in Response to BR Treatment

**Figure 2:**
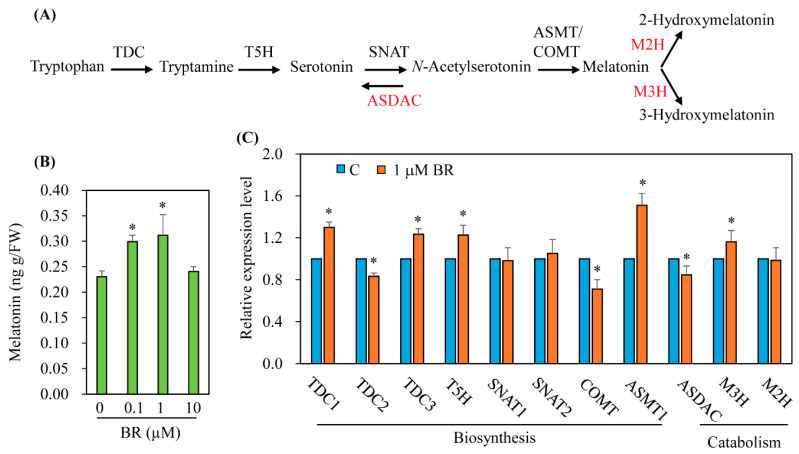
(**A**) Pathways of melatonin biosynthesis and catabolism. (**B**) Melatonin levels in rice seedlings treated with brassinosteroid in the absence of cadmium treatment. (**C**) Expression levels of transcripts encoding melatonin biosynthetic and catabolic genes. Seven-day-old rice seedlings were treated rhizosperically with various concentrations of epibrassinolide (BR) for quantification of melatonin or with 1 μM epibrassinolide (BR) for qRT-PCR for 24 h. The upper parts of leaves and stems were collected for HPLC quantification of melatonin and qRT-PCR analysis of various genes involved in melatonin synthesis and catabolism. The primer sequences were previously described [20]. C, water containing 0.1% ethanol. Asterisks (*) denote significant differences (*p* < 0.05; ANOVA, followed by Tukey’s HSD post hoc tests). C, water containing 0.1% ethanol; BR, epibrassinolide; *TDC1*, tryptophan decarboxylase 1 (AK069031); *TDC2* (AK103253); *TDC3* (Os08g0140500); *T5H*, tryptamine 5-hydroxylase (AK071599); *SNAT1*, serotonin *N*-acetyltransferase 1 (AK059369); *SNAT2* (AK068156); *COMT*, caffeic acid *O*-methyltransferase (AK064768); *ASMT1*, *N*-acetylserotonin O-methyltransferase (AK072740); *ASDAC*, *N*-acetylserotonin deacetylase (AK072557); *M3H*, melatonin 3-hydroxylase (AK067086); *M2H*, melatonin 2-hydroxylase (AK119413); *UBQ5*, ubiquitin 5 (Os03g13170).

To examine whether melatonin synthesis is induced by BR treatment in the absence of cadmium treatment, 7-day-old rice seedlings were challenged rhizosperically with varying concentrations of BR for 24 h, followed by quantification of melatonin using the upper parts of leaves and stems. As shown in Figure 2, melatonin synthesis increased with BR treatment at a concentration of 0.1 μM and reached a peak at 1 μM with a 40% increase compared to controls. Treatment with 10 μM BR failed to induce melatonin production, in contrast to cadmium treatment (Figure 1 and Figure 2). To determine whether the increase in melatonin synthesis by BR treatment is associated with genes involved in melatonin synthesis and catabolism, the shoot and stem tissues of 7-day-old rice seedlings challenged with 1 μM BR for 24 h were separated and harvested for total RNA extraction followed by quantitative real-time polymerase chain reaction (qRT-PCR) analysis using *UBQ5* as a reference gene. Transcript levels of melatonin biosynthetic genes, such as *tryptophan decarboxylase 1* (*TDC1*), *TDC3*, *tryptamine 5-hydroxylase* (*T5H*), and *N-acetylserotonin O-methyltransferase 1* (*ASMT1*), were significantly elevated by BR treatment, while the expression levels of the biosynthetic genes *TDC2* and *caffeic acid O-methyltransferase* (*COMT*) were downregulated (Figure 2C). Among catabolic genes, BR treatment inhibited the expression of *N-acetylserotonin deacetylase* (*ASDAC*), whereas the expression of *melatonin 3-hydroxylase* (*M3H*) was upregulated. The main difference in the effect on the increase in melatonin between GA and BR treatment was seen in *TDC1* expression, which was downregulated by GA treatment but upregulated by BR. In summary, the BR-mediated increase in melatonin is primarily related to the induction of melatonin biosynthetic gene transcription rather than the suppression of catabolic genes.

### 3.3. Melatonin Biosynthesis Was Severely Compromised in Transgenic Rice Plants with Various RNAis Downregulating the BR Biosynthesis-Related Genes DWARF4, DWARF11, and RAVL1

**Figure 3:**
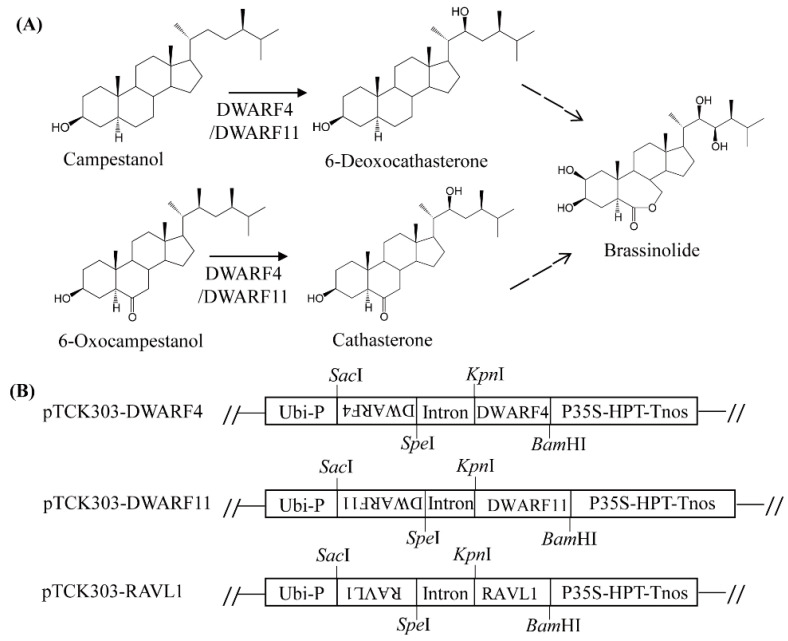
Brassinosteroid biosynthetic pathway and schematic diagram of binary vector. (**A**) Brassinosteroid biosynthesis and DWARF4 and DWARF11 enzymes. (**B**) Schematic diagram of pTCK303:DWARF4, pTCK303:DWARF11, and pTCK303:RAVL1 binary vectors. GenBank accession numbers of *DWARF4*, *DWARF11*, and *RAVL1* are AB206579, AK106528, and Os04g0581400. Ubi-P, maize ubiquitin promoter; P35S, 35 S cauliflower mosaic virus 35S promoter; HPT, hygromycin phosphotransferase; Tnos, nopaline synthase terminator.

**Figure 4:**
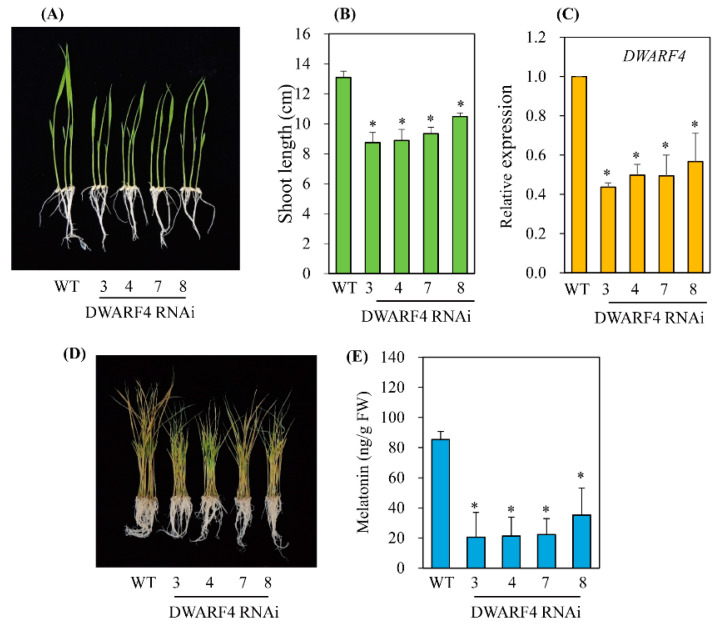
Generation of *DWARF4*-suppressed transgenic rice plants. (**A**) Phenotypes of 7-day-old wild-type (WT) and *DWARF4* RNAi transgenic (T_2_) rice seedlings. (**B**) Shoot length measurement. (**C**) qRT-PCR analyses of WT and transgenic (T_2_) lines. (**D**) Phenotypes of 7-day-old rice seedling after cadmium treatment. (**E**) Melatonin levels of WT and transgenic lines. Seven-day-old rice seedlings were challenged with 0.5 mM cadmium for 3 days for quantification of melatonin. Asterisks (*) indicate significant differences from the wild type (*p* < 0.05), as determined by Tukey’s HSD post-hoc tests.

**Figure 5:**
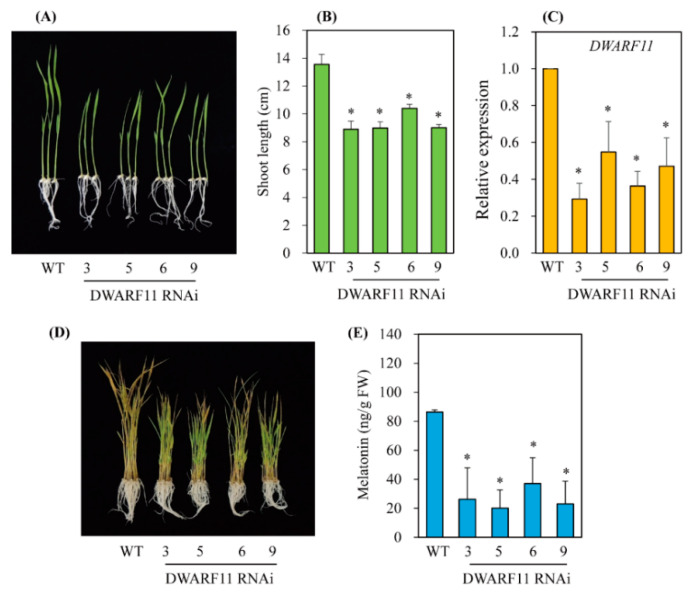
Generation of *DWARF11*-suppressed transgenic rice plants. (**A**) Phenotypes of 7-day-old wild-type (WT) and *DWARF11* RNAi transgenic (T_2_) rice seedlings. (**B**) Shoot length measurement. (**C**) Results of qRT-PCR analyses of WT and transgenic (T_2_) lines. (**D**) Phenotypes of 7-day-old rice seedlings after cadmium treatment. (**E**) Melatonin levels of WT and transgenic lines. The 7-day-old rice seedlings were challenged with 0.5 mM cadmium for 3 days for quantification of melatonin. Asterisks (*) indicate significant differences from the wild type (*p* < 0.05), as determined by Tukey’s HSD post hoc tests.

**Figure 6:**
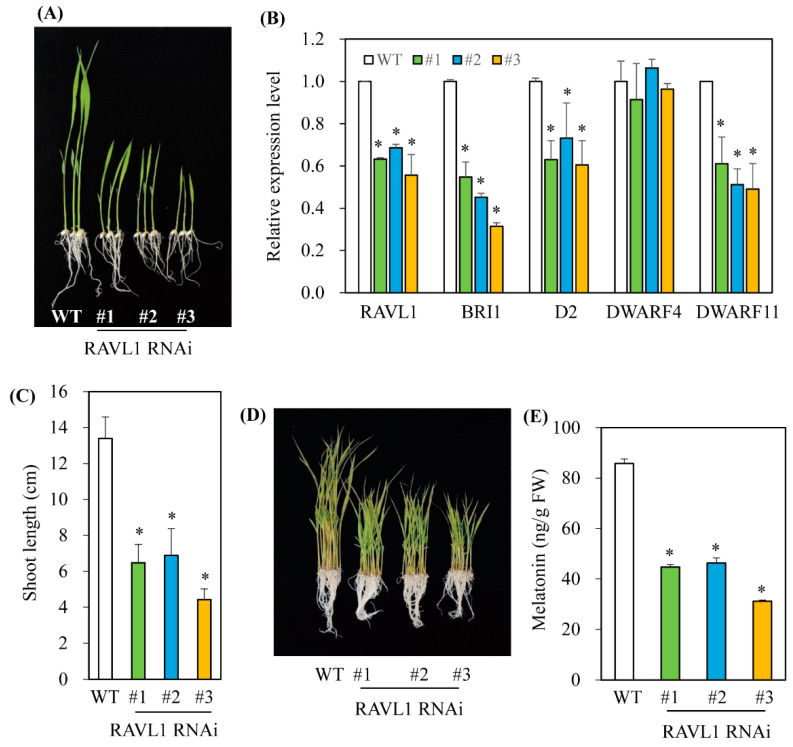
Generation of *RAVL1*-suppressed transgenic rice plants. (**A**) Phenotypes of 7-day-old wild-type (WT) and *RAVL1* RNAi transgenic (T_2_) rice seedlings. (**B**) Shoot length measurement. (**C**) qRT-PCR analyses of WT and transgenic (T_2_) lines. (**D**) Phenotypes of 7-day-old rice seedling after cadmium treatment. (**E**) Melatonin levels of WT and transgenic lines. The 7-day-old rice seedlings were challenged with 0.5 mM cadmium for 3 days for quantification of melatonin. Asterisks (*) indicate significant differences from the wild type (*p* < 0.05) as determined by Tukey’s HSD post hoc tests. GenBank accession numbers are Os04g0581400 (*RAVL1*), AK101085 (*BRI1*), XP_015755947 (*D2*), AK106528 (*DWARF11*), AB206579 (*DWARF4*), and Os03g13170 (*UBQ5*).

To investigate whether a decrease in BR leads to a reduction of melatonin biosynthesis, three BR biosynthesis-related genes were downregulated using an RNAi strategy (Figure 3). The two BR biosynthetic genes, *DWARF4* and *DWARF11*, which catalyze C-22 hydroxylation, the rate-limiting step of BR biosynthesis, have redundant functions, but show a 37% amino-acid identity [25]. The third gene, *RAV-like1* (*RAVL1*), is a transcription factor that binds BR biosynthetic genes, such as *DWARF11* and *ebisu dwarf* (*D2*), as well as the BR receptor *BRI1*, leading to increased BR synthesis [26,27]. All three RNAi transgenic rice seedlings exhibited suppression of transcripts in conjunction with a common dwarf phenotype (Figure 4, Figure 5 and Figure 6), a characteristic feature of BR deficiency. Melatonin levels were 25 ng/g fresh weight (FW) on average in both *DWARF4* and *DWARF11* RNAi rice seedlings, while the wild type produced melatonin at a level of 85 ng/g FW. *RAVL1* RNAi rice seedlings produced melatonin at a level of 38 ng/g FW on average, which was 2.2-fold lower than the wild type. *DWARF11*, *D2*, and *BRI1* transcripts were clearly suppressed in *RAVL1* RNAi rice seedlings, whereas *DWARF4* expression was not altered (Figure 6B), corroborating the involvement of *RAVL1* in both BR biosynthesis and signaling. Taken together, these observations are consistent with the results of exogenous BR treatments, highlighting the role of BR as an elicitor of melatonin biosynthesis in vitro and in vivo, similar to GA [20].

### 3.4. BR Induced Melatonin Synthesis in a GA-Independent Manner

**Figure 7:**
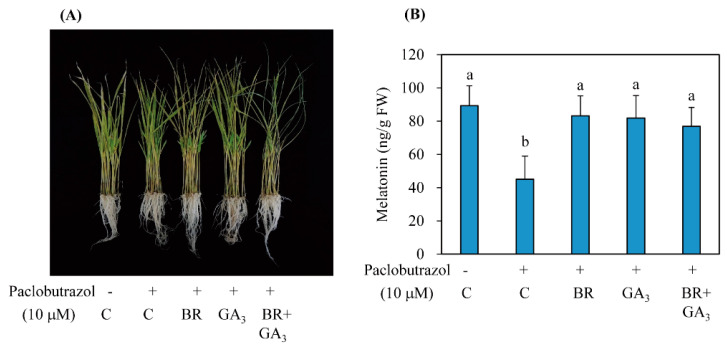
Restoration of melatonin synthesis by BR treatment under conditions of inhibition of GA biosynthesis. (**A**) Phenotypes of 7-day-old wild-type (WT) rice seedling treated with paclobutrazol together with either BR or GA_3_ alone or in combination for 24 h. The resulting rice seedlings were subjected to cadmium treatment (0.5 mM) for 3 days under conditions of continuous light for quantification of melatonin. (**B**) Melatonin quantification. The different letters denote significant differences (*p* < 0.05) as determined by Tukey’s HSD post hoc tests from the control (C). PB, paclobutrazol (10 μM); C, water containing 0.1% ethanol; BR, epibrassinolide (1 μM); GA_3_, gibberellic acid 3 (10 μM).

**Figure 8:**
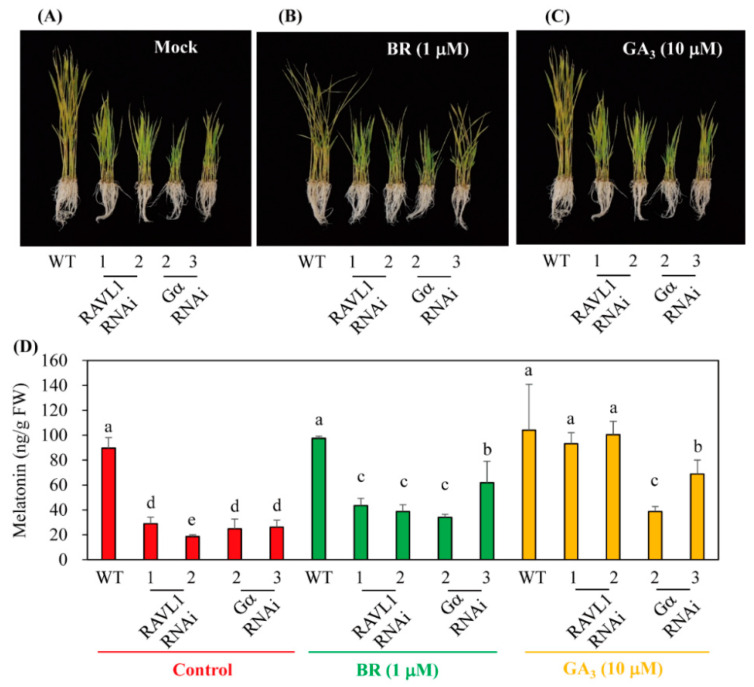
Effects of exogenous BR and GA in *RAVL1* and *Gα* RNAi transgenic rice seedlings. (**A–C**) Phenotypes of 7-day-old rice seedlings after cadmium treatment in response to exogenous BR and GA_3_ treatments. (**D**) Melatonin levels of control-, BR-, and GA-treated rice seedlings. Seven-day-old wild-type (WT) and RNAi rice seedlings were challenged with either BR or GA for 24 h. The resulting rice seedlings were treated with 0.5 mM cadmium for 3 days under conditions of continuous light at 28 °C followed by HPLC quantification of melatonin. Different letters denote significant differences (*p* < 0.05), as determined by Tukey’s HSD post hoc tests. Mock, water containing 0.1% ethanol; BR, 1 μM epibrassinolide; GA_3_, 10 μM gibberellic acid 3.

The 7-day-old rice seedlings were challenged with 10 μM paclobutrazol (PB), a GA biosynthesis inhibitor, together with either BR or GA alone or BR + GA to determine whether PB-mediated melatonin decrease could be reversed. As shown in Figure 7, the PB-mediated reduction of melatonin was fully reversed by either BR or GA alone and in combination. These data suggest that BR-induced melatonin induction is not dependent on GA-induced melatonin synthesis. The synergistic increase in melatonin level by combined treatment with BR and GA compared to either individual treatment alone has not been observed. Unlike cell elongation in which BR acts as an upstream regulator of GA biosynthesis [21], the increase in melatonin level associated with BR treatment did not require the downstream GA regulator. These pharmacological complementation experiments were further verified in *RAVL1* and *Gα* RNAi transgenic rice plants. Melatonin decreases in these transgenic plants were partially ameliorated by exogenous BR treatment because these genes are associated with the BR signaling pathway (Figure 8). However, the decrease in melatonin level in the *RAVL1* RNAi transgenic rice was fully reversed by exogenous GA treatment, but only partially reversed in the *Gα* RNAi transgenic rice plants. These observations clearly indicate that BR and GA act independently to induce melatonin synthesis.

## 4. Discussion

The first report of crosstalk between BR and melatonin came from transgenic rice plants with downregulation of *serotonin N-acetyltransferase 2* (*SNAT2*), the penultimate gene in the melatonin biosynthesis pathway [33]. The *SNAT2*-suppressed RNAi rice plants showed a BR-deficient phenotype exhibiting semi-dwarf with erect leaves in conjunction with BR decrease. This indicated that melatonin decrease is related to BR decrease, while *SNAT2* overexpression did not result in an increase in BR, suggesting an indirect effect of melatonin on BR synthesis, similar to the GA decrease in *SNAT1* knockout *A*. *thaliana* mutant [8,34]. In addition to the effect of melatonin on the levels of various plant hormones [13], we recently reported that GA acts as an endogenous elicitor to induce melatonin synthesis [20]. One possible explanation for GA-induced melatonin synthesis is that GA-mediated growth promotion is susceptible to many stresses, including H_2_O_2_ [35], which may be counteracted by an increase in melatonin, as melatonin is a potent antioxidant as well as a signaling molecule with roles in protecting plants from a diverse array of abiotic and biotic stresses by way of the induction of many antioxidant enzymes and their defense-related genes [1,3,36,37,38,39].

**Figure 9:**
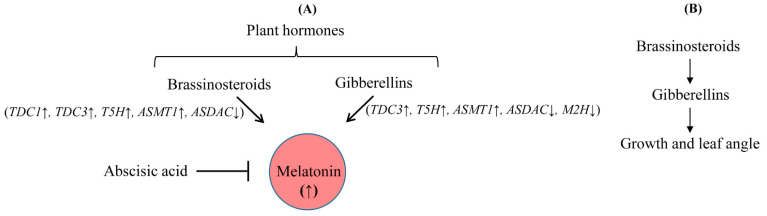
Proposed model for melatonin biosynthesis by endogenous hormones. (**A**) Brassinosteroids (BR)-induced melatonin increase in a gibberellins (GA)-independent manner and (**B**) BR-induced GA-dependent regulation of growth and leaf angle. GA and BR induce melatonin production independently, whereas abscisic acid inhibits melatonin production. The induction patterns of genes responsible for melatonin biosynthesis and metabolism were slightly different between hormone treatments. BR treatment induced several genes involved in melatonin biosynthesis, including *TDC1*, *TDC3*, *T5H*, and *ASMT1*, whereas GA induced *TDC3*, *T5H*, and *ASMT1*. *ASDAC* and *M2H* were downregulated by GA treatment, while only *ASDAC* was downregulated by BR treatment. Unlike BR and GA, abscisic acid inhibits melatonin synthesis [20].

Interestingly, it was reported that BR regulates cell elongation by modulating GA biosynthesis in rice [21] and *A*. *thaliana* [22], suggesting that BR treatment may also induce melatonin synthesis indirectly via an increase in GA. In this study, BR treatment induced melatonin synthesis in a dose-dependent manner, similar to GA, while the other hormones, ethylene, BA, and IAA, failed to induce melatonin synthesis (Figure 1). To gain direct genetic evidence, we used RNAi for three BR synthesis- and signaling-related genes, i.e., *DWARF4*, *DWARF11*, and *RAVL1*. Transgenic rice plants with RNAi-mediated downregulation of these genes showed decreased synthesis of melatonin, indicating that BR acts as an endogenous elicitor in plants. The greatest decline in melatonin synthesis was observed in the *DWARF4* RNAi rice followed by *DWARF11* RNAi and *RAVL1* RNAi lines. Melatonin synthesis was markedly decreased in the presence of paclobutrazol, a GA biosynthesis inhibitor, while either exogenous BR or GA treatment fully recovered melatonin synthesis. These observations suggested that BR-mediated melatonin induction is independent of GA, with both acting as an independent elicitor of melatonin synthesis (Figure 9). These data were further confirmed by the observation that GA treatment of the *RAVL1* RNAi rice seedlings fully recovered melatonin synthesis comparable to wild type, but only partially recovered melatonin synthesis in *Gα* RNAi rice seedlings. The partial recovery of melatonin synthesis in the *Gα* RNAi line by either GA or BR indicated that Gα is involved in the signaling processes of both GA and BR.

Commensurate with the induction of melatonin biosynthetic genes in response to GA, BR treatment induced many genes responsible for melatonin biosynthesis, including *TDC3* and *T5H*. In addition, *M2H* was suppressed in response to BR, similar to GA. However, differential gene expression between BR and GA treatments was also observed in *TDC1*, *TDC2*, *SNAT1*, *SNAT2*, and *M3H*. Taken together, these results indicate that BR acts as an elicitor for induction of melatonin synthesis, similar to GA [23]. Both BR and GA seem to play independently roles in melatonin synthesis (Figure 9A) unlike the BR-mediated GA increase pathway in growth and leaf angle (Figure 9B). The results presented here suggest that melatonin synthesis is positively correlated with endogenous levels of either GA or BR in plant cells.

## 5. Conclusions

Melatonin is a pleiotropic molecule with various functions in plant growth and development as well as in defense responses to abiotic and biotic stresses. Although there have been many reports of changes in plant hormones by melatonin treatment, there have been few studies of the effects of plant hormones on melatonin biosynthesis. Here, we demonstrated that BRs play essential roles in the induction of melatonin synthesis, similar to GA. Our findings indicate that melatonin seems to function in concert with BR and GA during plant growth and development. As both BR and GA are commonly involved in cell elongation and growth, which are accompanied by increased susceptibility to various abiotic stresses [35,40,41], the concomitant increases in melatonin level by BR and GA may counteract these adverse effects due to increases in either GA [40] or BR [41] by increasing protein quality control and antioxidant activity exerted by melatonin [1,3,5,9]. Further in-depth studies of the precise underlying mechanisms and interplay between plant hormones and melatonin will facilitate the discovery of new functions of melatonin in plant growth and development as well as in defense responses to stressors.

## Data Availability

Data presented in this study are available within the article.
